# Supplementary material for: Accelerating the Laboratory Testing Capacity through Saliva Pooling Prior to Direct RT-qPCR for SARS-CoV-2 Detection
Source: Diagnostics (Basel). 2022 Dec 14;12(12):3160. doi: 10.3390/diagnostics12123160 (PMC9777453; doi:10.3390/diagnostics12123160)
Supplement: Supplementary file 1 [file diagnostics-12-03160-s001.zip › diagnostics-2011624-supplementary.pdf]

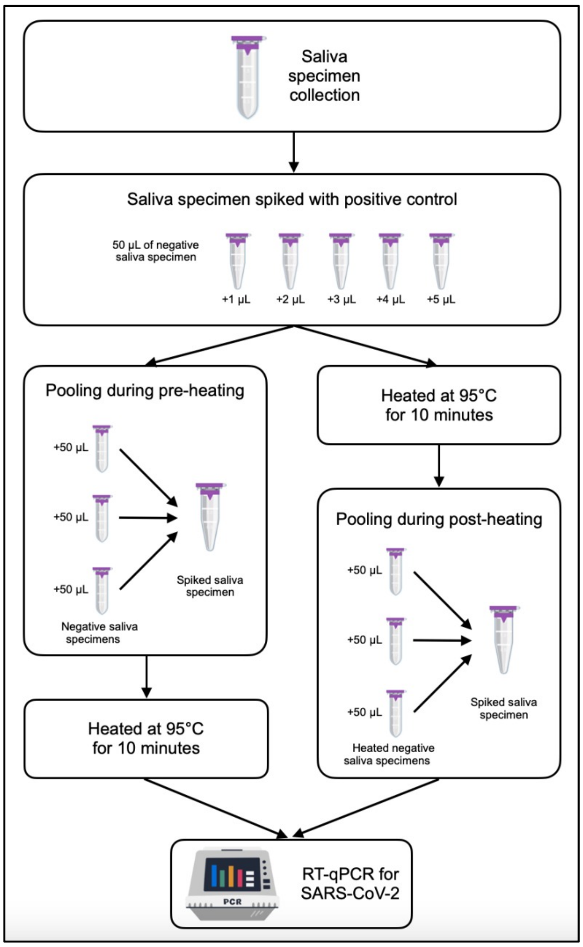

Supplementary Figure S1. Schematic workflow of the timing validation in which the pooling method was conducted (pre- and post-heating). Saliva specimens were collected and spiked with 1, 2, 3, 4 or 5 µL positive control. On pre-heating pooling, 50 µL of three saliva specimens were combined with 50 µL of a spike-in negative saliva specimen, followed by inactivation through heating. Meanwhile, during post-heating, each specimen was heated separately prior to pooling using the same specimen combination as pre-heating. Pools were processed using RT-qPCR to detect SARS-CoV-2.

Supplementary Table S1. The commercial RT-qPCR kits specification used in this study. Each kit has specific target genes, internal control, limit of detection (LOD), reaction volume, template volume, number of cycles, and cycle cut-off, which might have similar or different values.

Kit Name	Abbreviation*	Target Genes – Reporter Dye	Internal Control – Reporter Dye	LOD (copies/mL)	Reaction Volume (µL)	Template Volume (µL)	No. of Cycles	Cycle Cut–off
Multiple Real-Time PCR Kit for Detection of 2019-CoV	xABT	ORF1ab – FAM<br>N gene – VIC<br>E gene - FAM	Pseudovirus – Cy5	200	15	5	45	38
Novel Coronavirus (COVID-19) Nucleic Acid Detection Kit	Ardent	ORF1ab – FAM<br>N gene – VIC	RNase P – Cy5	400	15	5	45	40

(PCR-fluorescent probe)								
Standard M nCoV Real-Time Detection kit	SD Biosensor	ORF1ab – FAM<br>E gene – HEX/VIC	Internal control A (Pseudovirus) – Cy5	250	20	10	40	36
Real-Q 2019-nCoV Detection Kit	BioSewoom	RdRP gene – FAM<br>E gene – HEX/VIC	HRP – Cy5	3125	20	5	40	38
SARS-CoV-2 Nucleic Acid Detection Kit (Real-time RT-PCR Method)	Tianlong	RdRP gene – FAM<br>N gene – HEX/VIC	GADP – Cy5	200	20	5	45	40
Detection Kit for	Da An Gene	N gene – FAM	RNase P – Cy5	500	25	5	45	40

2019 Novel Coronavirus (2019-nCoV)	ORF1ab – VIC
* Based on kit name	

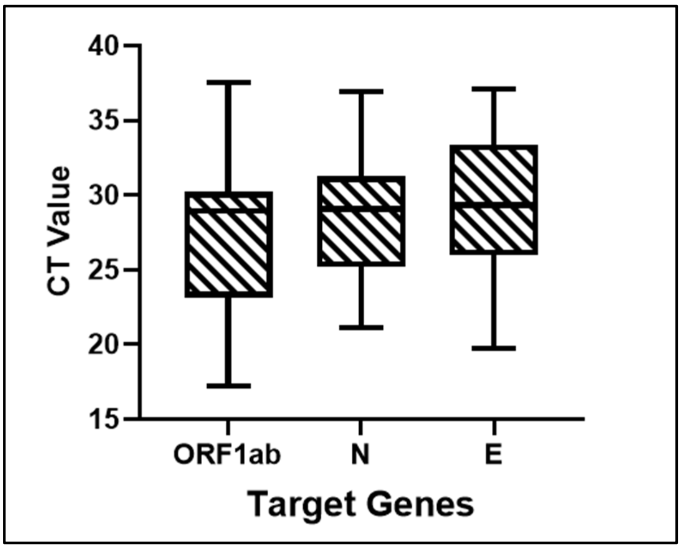

Supplementary Figure S2. Viability test results using XABT kit. The XABT kit detected 3 gene targets. Twenty four samples that were tested positive before storage, remained positive after seven months of storage at -80°C.
